# Epidemiology of mpox: Focus on men with HIV

**DOI:** 10.1016/j.heliyon.2023.e22129

**Published:** 2023-11-08

**Authors:** Nadim Sharif, Shuvra Kanti Dey

**Affiliations:** Department of Microbiology, Jahangirnagar University, Savar, Dhaka-1342, Bangladesh

**Keywords:** Mpox, HIV, Risk group, Men

## Abstract

The 2022 mpox outbreak is the first ever report of worldwide spread of cases. Integrated knowledge on the epidemiology and clinical characteristics of mpox are limited. This study was conducted to shed light on the epidemiology of 2022 mpox outbreak. We found that men were the most infected sex (90–100 % cases). The highest prevalence of mpox infection (70 %) was found among men aged between 30 and 40 years. Pre-existing HIV was reported among 24–100 % of mpox positive cases. About 90–100 % of the cases have been disproportionately found among group of men with specific sexual practice, namely, men who have sex with men (MSM). Case fatality rate of 2022 mpox outbreak varied between 1 and 10 %. Studies on the relationship of HIV with mpox outcomes are limited. This study will add knowledge on the epidemiology of 2022 mpox outbreak.

## 1 Introduction

The 2022 Mpox (previously monkeypox) outbreak has transmitted across 110 countries with case number surpassed 84 075 on January 11, 2023 [1,2]. No historical records of mpox have been reported from majority of the localities (94 %, 103 of 110) before the 2022 outbreak. Nearly 99 % cases and fatalities are reported from these non-endemic regions [1,2]. The highest number of cases (nearly 30 000) have been recorded in USA with 20 deaths. Historically mpox was isolated in 1958 from outbreaks in cynomolgus monkeys with smallpox like symptoms in Copenhagen [[1], [2], [3]]. During 1970s, several outbreaks were reported from monkeys in the USA and the Netherlands. The first symptomatic case of human mpox was documented in 1970 during smallpox surveillance in Democratic Republic of Congo from a 9 months old child followed by another six cases in young children during 1970–1971 in West Africa [[1], [2], [3], [4], [5]].

Patients with mpox infection develop characteristics symptoms [[4], [5], [6], [7], [8], [9]]. The most common symptoms include fever, multiple popular lesions, vesiculopustular lesions, and ulcerative lesions on the body and face and lymphadenopathy [[3], [4], [5], [6], [7], [8], [9], [10]]. Case fatality rate may vary between 1 and 10 % depending on the clade of mpox infection. Severe illness and other complications like encephalitis, pneumonitis and secondary infection are higher in children, elderly and HIV infected patients [[3], [4], [5], [6], [7], [8], [9], [10]].

Integrated studies on the epidemiology, clinical characteristics and transmission route of mpox are scarce. Only few studies are available with limited knowledge of the previous sporadic outbreaks. Recently, as case number are increasing, studies focusing on clinical features and transmission are getting highlighted to understand the baseline of the outbreaks. This study was conducted to create an integrated insights about the epidemiology, route of transmission in human body along with clinical data of mpox.

## 2 Method

Findings from different original research works were included to conduct the study. We included articles from searching in MEDLINE (through PubMed), EMBASE, Web of Science, Scopus, the Internet Library sub-Saharan Africa (ilissAfrica), African Journals Online (AJOL), The New England Journal of Medicine (NEJM) and The Lancet with no restriction on language and place. All published articles and scientific writings till January 20, 2023 were included in this study. The notable search term included Monkeypox, Mpox, Monkey pox, MPXV, Epidemiology of mpox, Clinical characteristics of Mpox, Cases of Monkeypox, Transmission of mpox, Transmission of Monkeypox, “variole simienne”, and “variole du singe” and combination of these terms. Additionally, we conducted search on the grey literature and Google Scholar. These sources included databases from CDC (Centers for Disease Control and Prevention, USA), ECDC (European Centers for Disease Control and Prevention) WHO (the World Health Organization), Epicentre, ProMed, CDC of Nigeria, CDC of Africa, and African Field Epidemiology Network. Total number of cases was determined for each decade by summation of the reported cases per clades. Pooled Statistical analyses were conducted by using SAS version 9.4.

## 3 Epidemiology of 2022 mpox outbreak

Community transmission of 2022-mpox outbreaks has been found disproportionately among men who have sex with men (MSM) gay, and bisexual in several countries including the new hotspot, USA [1,2] followed by Brazil, Spain and France [2]. The natural history of mpox is still unknown and the recent studies on epidemiology and transmission of 2022-mpox outbreak suggests unusual features than previous sporadic and local outbreaks. We analyzed the scientific evidences to add knowledge about the risk group and epidemiology of 2022-mpox outbreaks.

Firstly, the reported cases from Americas, Europe and other severely affected areas were most frequently found among men (90–100 %) (Table 1) [2,3,5,[8], [9], [10], [11], [12], [13],^14^,[15], [16], [17], [18], [19]]. Epidemiological and clinical data suggested that nearly half of the reported cases of mpox have previous HIV infection (Table 1). The prevalence of HIV positive cases varied from 24 to 100 % among the mpox patients in the ongoing outbreak. In most of the studies with the prevalence of HIV positive patients were between 30 and 55 % [[2], [3], [4], [5], [6],^8^,^9^,^11^,[14], [15], [16], [17], [20],^19^]. However, in two studies, the prevalence was 100 % [12,21]. We rarely found studies directly relating HIV as a risk factor of mpox infection. However, it is evident that people living with HIV have weaker immunity than without HIV. Accordingly, prognosis of mpox and health outcomes among HIV-positive and HIV-negative cases are required to be analyzed extensively to predict the impacts. Case fatality rate of patients co-infected with mpox-HIV was higher than patients with mpox alone. Previous case series analysis in 2017 in Nigeria [22] have also found higher case fatality rate among the HIV co-infected cohort. We have also found reports of bacterial secondary infection in the genitals of mpox-HIV co-infected patients, which resulted in hospitalization and prolonged illness in this 2022 outbreak [10].

**Table 1:** Epidemiological characteristics of 2022-mpox outbreak.

Study	Participants	HIV-positive	Specific behaviors	Age Median (IQR)	Skin∗	Anogenital	Semen	Saliva
			MSM		MPXV DNA prevalence	MPXV DNA prevalence	MPXV DNA prevalence	MPXV DNA prevalence
European Region (ECDC, 2023) [2]	25 471	3927/10 366 (38 %)	96 %	31–40+	100 %‡	NA	NA	NA
Spain (Hernaez et al., 2022) [3]	44	23/44 (52 %)	94 %	35 (11·3)	NA	NA	NA	85 %
Italy (Agrati et al., 2022) [4]	17	7/17 (41 %)	100 %	39·5 (33·5–45·25)	100 %	NA	NA	NA
France (Mailhe et al., 2022) [5]	264	73/256 (29 %)	95 %	35 (30–41)	98 %	NA	NA	NA
Spain (Tarín-Vicente et al., 2022) [6]	181	72/181 (40 %)	92 %	37 (31–42)	99 %	78 %	NA	NA
Italy (Raccagni et al., 2022) [7]	36	15/36 (42 %)	100 %	34 (29–36.5)	100 %§	.	61 %	NA
15 countries (Angelo et al., 2022)∗∗ [8]	226	92/209 (44 %)	99 %	37 (32–43)	100 %	NA	NA	NA
Spain (Peiró-Mestres et al., 2022) [9]	12	4/12 (33 %)	98 %	38.5 (32–52) +	100 %	92 %	78 %	100 %
UK (Patel et al., 2022) [10]	197	70/195 (35.9 %)	99.5 %	38	100 %^‡^	NA	NA	NA
16 countries (Thornhill et al., 2022)† [11]	528	218/528 (41 %)	98 %	38 (18–69) +	100 %‡	.	91 %	NA
Italy (Lapa et al., 2022) [12]	1	1/1 (100 %)	100 %	39	NA	NA	100 %	NA
Germany (Hoffmann et al., 2022) [13]	546	256/546 (47 %)	100 %	39 (20–67) +	100 %‡	NA	NA	NA
Spain (Vivancos-Gallego et al., 2022) [21]	25	25/25 (100 %)	100 %	39.5 (33–46)	91 %	90 %	NA	NA
Portugal (Duque et al., 2022) [14]	27	14/27 (52 %)	95 % (18/19)	33 (22–51)	100 %‡	NA	NA	NA
Spain (Echevarría et al., 2023) [20]	49	15/49 (31 %)	96 %	37.6#	100 %^‡^	80 %	NA	NA
USA (Philpott et al., 2022) [15]	1195	136/334 (41 %)	94 %	35 (30–41)	100 %‡	NA	NA	NA
Spain (Iñigo Martínez et al., 2022) [16]	508	225/508 (42 %)	93 %	35 (18–67) +	100 %‡	NA	NA	NA
UK(Girometti, N., et, 2022) [17]	54	13/54 (24 %)	100 %	41 (34–45)	100 %^‡^	NA	NA	NA
Spain (Orviz, E. et al., 2022) [18]	48	19/48 (40 %)	87.5 %	35 (29–44)	100 %‡	NA	NA	NA
France (Palich et al., 2022) [19]	50	22/50 (44 %)	98 %	34 (29–40)	88 %	71 %	54 %	NA

IQR- Interquartile range.

∗ Includes perianal skin.

† Argentina, Australia, Belgium, Canada, Denmark, France, Germany, Israel, Italy, Mexico, Portugal, Spain, Switzerland, The Netherlands, UK, and USA.

∗∗ Argentina, Belgium, Canada, Denmark, France, Germany, Israel, Portugal, South Africa, Spain, Sweden, Romania, The Netherlands,UK, and USA.

+ Range in years.

# Mean age.

‡ Skin or anogenital samples combined.

§ Either skin, anogenital, or oropharyngeal samples combined.

Secondly, the mpox infection was most prevalent among people with median age of 30–40 years (Table 1) [13,[14], [15], [16], [17], [20],^19^]. The reported number of cases in 2022-mpox outbreaks among children (below 16 years) and elderly persons (above 60 years) are significantly lower than previous outbreaks. During the early period (May–July 2022), proportionate incidence of mpox was high among white people (nearly 67 %), which was replaced by black or African American and Hispanic or Latino with time (from 6 % to 50 %) [1,2,[5], [6], [7], [8], [9],^11^,^16^]. We found disproportionate distribution of cases among the mentioned age groups, which remained nearly the same during the outbreaks. The exact reasons and transmission need to be better analyzed and described for generalize statement and defining risk group. There is evidence of asymptomatic cases of mpox, which suggest that underreporting is continuously occurring [13,21,20,15,17,19]. A vigilance and rapid surveillance with wide scopes are required to include the asymptomatic cases and those unwilling to report without notable symptoms. The existing data and knowledge partially support that, men aged between 30 and 40 years with HIV infection, come in close contact with diseased patients, have higher frequency of getting mpox infection [1,3,9,[11], [12], [13], [14], [21],^15^,^17^].

Finally, a lot of studies on the route and sources of transmission of 2022-mpox outbreaks have been conducted and identified few probable routes responsible for higher frequency of the cases. The characteristics muco-cutaneous lesions of mpox have been most frequently isolated from the genitals, anogenital and perineal areas than other organs of the body, which was higher in face, arms, and legs in previous outbreaks [2,[4], [5], [6], [7],[9], [10], [11],^21^,^16^,^17^]. Recent studies have reported higher prevalence (54–100 %) and concentration of mpox DNA in semen sample of the infected men [7,9,11,[13], [21], [14], [20], [15], [16], [17], [18], [19], [22]]. Several studies have found that isolated DNA of mpox from the lesions of anogenital skin and semen have capability to infect cell lines like Vero E6 cells and produce cytopathic effects [12,13]. However, these are preliminary studies to define and characterize the transmission route of 2022-mpox outbreaks. Interestingly, 90–100 % of the cases have been disproportionately reported among group of people with specific sexual practice, namely, men who have sex with men (MSM) (Table 1). Data and findings from numerous studies support the sexual transmission of 2022-mpox outbreaks among MSMmen [3,4,6,8,[13], [21], [14], [20], [15], [16], [17], [18], [19]]. Based on these findings can we categorize 2022-mpox as a sexually transmitted disease? Actually, the preliminary data supports further extensive investigation on the mechanism of transmission of mpox among the infected, from the infected to the healthy susceptible via sexual interactions or other routes [13,[14], [15], [16], [17], [18], [19], [20]]. Till now data are scarce about how mpox DNA mixed with semen samples. The main limitation of this study is that it couldn't add all the available data and necessary information.

## 4 Conclusion

In conclusion, the public health measures, surveillance and preventive measures of mpox should highlight on the MSM men with HIV and those came in contact with them recently. Awareness should be raised among them to immediate reporting to the local health centers if symptoms arise and avoid direct-contact with their partners. This study also finds a significant gap on the epidemiological characteristics of the minor groups and asymptomatic patients. In addition, genomic characterization of mpox, detailed clinical and epidemiological analysis of asymptomatic cases, and minor number of cases outside MSM men are required to understand the complete scenario of risk factors, risk group and appropriate measures to prevent the outbreak.

## Data availability

All the necessary data will be made available on request.

## CRediT authorship contribution statement

**Nadim Sharif:** Conceptualization, Formal analysis, Investigation, Methodology, Project administration, Software, Supervision, Writing – original draft, Writing – review & editing. **Shuvra Kanti Dey:** Investigation, Resources, Software, Supervision, Writing – review & editing.

## Declaration of competing interest

The authors declare that they have no known competing financial interests or personal relationships that could have appeared to influence the work reported in this paper.

## References

[1] (2022). Centers for disease Control and prevention. Monkeypox outbreak global map.

[2] (2022. January 21). European Centers for Disease Prevention and Control; World Health Organization Regional Office for Europe. Joint ECDC-WHO Reginal Office for Europe Monkeypox Surveillance Bulletin.

[3] Hernaez B., Muñoz-Gómez A., Sanchiz A. (2022). Monitoring monkeypox virus in saliva and air samples in Spain: a cross-sectional study. Lancet Microbe.

[4] Agrati C., Cossarizza A., Mazzotta V. (2023). Immunological signature in human cases of monkeypox infection in 2022 outbreak: an observational study. Lancet Infect. Dis..

[5] Mailhe M., Beaumont A.L., Thy M. (2023). Clinical characteristics of ambulatory and hospitalized patients with monkeypox virus infection: an observational cohort study. Clin. Microbiol. Infect..

[6] Tarín-Vicente E.J., Alemany A., Agud-Dios M. (2022). Clinical presentation and virological assessment of confirmed human monkeypox virus cases in Spain: a prospective observational cohort study. Lancet.

[7] Raccagni A.R., Candela C., Mileto D. (2022). Monkeypox infection among men who have sex with men: PCR testing on seminal fluids. J. Infec.

[8] Angelo K.M., Smith T., Camprubí-Ferrer D. (2023). Epidemiological and clinical characteristics of patients with monkeypox in the GeoSentinel Network: a cross-sectional study. Lancet Infect. Dis..

[9] Peiró-Mestres A., Fuertes I., Camprubí-Ferrer D. (2022). Frequent detection of monkeypox virus DNA in saliva, semen, and other clinical samples from 12 patients, Barcelona, Spain, May to June 2022. Euro Surveill..

[10] Patel A., Bilinska J., Tam J.C. (2022). Clinical features and novel presentations of human monkeypox in a central London centre during the 2022 outbreak: descriptive case series. bmj.

[11] Thornhill J.P., Barkati S., Walmsley S. (2022). Monkeypox virus infection in humans across 16 countries—april–June 2022. N. Engl. J. Med..

[12] Lapa D., Carletti F., Mazzotta V. (2022). Monkeypox virus isolation from a semen sample collected in the early phase of infection in a patient with prolonged seminal viral shedding. Lancet Infect. Dis..

[13] Hoffmann C., Jessen H., Wyen C. (2023). Clinical characteristics of monkeypox virus infections among men with and without HIV: a large outbreak cohort in Germany. HIV Med..

[14] Duque M.P., Ribeiro S., Martins J.V. (2022). Ongoing monkeypox virus outbreak, Portugal, 29 April to 23 May 2022. Euro Surveill..

[15] Philpott D. (2022). Epidemiologic and clinical characteristics of monkeypox cases—United States, May 17–July 22, 2022. MMWR. Morbidity and Mortality Weekly Report.

[16] Iñigo Martínez J., Gil Montalbán E., Jiménez Bueno S. (2022). Monkeypox outbreak predominantly affecting men who have sex with men, Madrid, Spain, 26 April to 16 June 2022. Euro Surveill..

[17] Girometti N., Byrne R., Bracchi M. (2022). Demographic and clinical characteristics of confirmed human monkeypox virus cases in individuals attending a sexual health centre in London, UK: an observational analysis. Lancet Infect. Dis..

[18] Orviz E., Negredo A., Ayerdi O. (2022). Monkeypox outbreak in Madrid (Spain): clinical and virological aspects. J. Infect..

[19] Palich R., Burrel S., Monsel G. (2023). Viral loads in clinical samples of men with monkeypox virus infection: a French case series. Lancet Infect. Dis..

[20] Echevarría A.G., Leal R.P., Domenech Á.M. (2023). Clinical and demographic features of 49 patients with human monkeypox virus confirmed infection in a tertiary care centre in Valencia, Spain: a descriptive study. Sex. Transm. Dis..

[21] Vivancos-Gallego M.J., Sánchez-Conde M., Rodríguez-Domínguez M. (2022). Human monkeypox in people with HIV: transmission, clinical features, and outcome. Open Forum Infect. Dis..

[22] Sharif N., Sharif N., Alzahrani K.J. (2023). Molecular epidemiology, transmission and clinical features of 2022‐mpox outbreak: a systematic review. Health Sci. Rep..

